# Online Learning State Evaluation Method Based on Face Detection and Head Pose Estimation

**DOI:** 10.3390/s24051365

**Published:** 2024-02-20

**Authors:** Bin Li, Peng Liu

**Affiliations:** School of Computer Science, Northeast Electric Power University, Jilin 132011, China; pengliujilin@163.com

**Keywords:** online learning state evaluation, face detection, head pose estimation

## Abstract

In this paper, we propose a learning state evaluation method based on face detection and head pose estimation. This method is suitable for mobile devices with weak computing power, so it is necessary to control the parameter quantity of the face detection and head pose estimation network. Firstly, we propose a ghost and attention module (GA) base face detection network (GA-Face). GA-Face reduces the number of parameters and computation in the feature extraction network through the ghost module, and focuses the network on important features through a parameter-free attention mechanism. We also propose a lightweight dual-branch (DB) head pose estimation network: DB-Net. Finally, we propose a student learning state evaluation algorithm. This algorithm can evaluate the learning status of students based on the distance between their faces and the screen, as well as their head posture. We validate the effectiveness of the proposed GA-Face and DB-Net on several standard face detection datasets and standard head pose estimation datasets. Finally, we validate, through practical cases, that the proposed online learning state assessment method can effectively assess the level of student attention and concentration, and, due to its low computational complexity, will not interfere with the student’s learning process.

## 1. Introduction

Online learning has gradually become a popular form of education due to its unrestricted nature of space and time. Teachers in online education are unable to monitor students’ learning status in real time, resulting in the inability to guarantee their learning outcomes. Therefore, designing a learning-state-monitoring method that can run on mobile devices can compensate for the insufficient supervision of students in online education.

Existing learning state assessment methods mainly include two categories: bioinformatics-based methods and image-based methods. Methods based on bioinformatics use instruments to detect a person’s bioinformatics (such as electrocardiograms, electroencephalograms, and breathing, etc.) to analyze their level of individual attention. Avila et al. [1] detected the pulse of subjects using an optical heart rate sensor and established 12 pulse templates to match with standard electrocardiograms. Zhang et al. [2] proposed a method for evaluating instantaneous attention through electroencephalography (EEG) and validated the feasibility of predicting sustained attention fluctuations using this method. Sharma et al. [3] used radio frequency sensors to detect heartbeat and respiration, and evaluated attention through heartbeat and respiration.

The collection of biological information requires additional equipment support, and in real life, it is difficult to require users to equip these devices. Compared to bioinformatics-based methods, image-based learning state supervision methods often only require cameras and have the characteristics of low cost and convenient use. Palinko et al. [4] developed an eye-tracking system and demonstrated that eye tracking can more accurately estimate a person’s attention focus. Andrea et al. [5] believed that, in real-life scenarios, ordinary cameras detecting human eye movements and line of sight are easily affected by the environment and require special equipment support. Therefore, they proposed inferring attention based on human head posture, and demonstrated through experiments that head posture can robustly reflect attention focus. Li et al. [6] proposed a method to analyze students’ attention through multimodal inputs of cameras and computer mice. Combining students’ expressions and operating behaviors can more accurately evaluate students’ attention than single-mode data. The methods based on head pose estimation mentioned above are less susceptible to environmental interference. Therefore, this article proposes a method for evaluating student attention through facial detection and head pose estimation. This method can complete real-time assessments of student learning status on mobile devices with low computing power. The contributions of the article are as follows:

(1) We propose a face detection network based on ghost module and parameter-free attention (GA-Face). GA-Face reduces the number of parameters and computation required to generate redundant features in the feature extraction network through the ghost module [7]. GA-Face also uses the parameter-free attention module SimAM [8] to focus the network on important features.
(2) We propose a head pose estimation network, DB-Net, based on dual-branch attention. DB-Net has two feature extraction branches: color and grayscale. DB-Net uses the ghost module to enhance its ability to extract deep semantic information and reduce its parameter count. DB-Net uses convolutional fusion methods to prevent the generation of useless features.
(3) The real-time detection of student head position and posture on mobile devices using GA-Face and DB-Net, and the scoring of student attention using learning state evaluation algorithms.

## 2. Related Work

### 2.1. Facial Detection

In facial detection tasks, the detection performance of deep learning methods is far superior to that of traditional methods. With the development of deep learning technology, 3D object detection methods [9] that can provide more accurate target information have emerged. However, this method is not suitable for mobile devices with lower computing power. Deep-learning-based methods used for 2D object detection can be further divided into anchor-based methods and anchor-free methods. The R-CNN series [10] algorithm, SSD [11], and RetinaNet [12] are representative anchor-based detection algorithms. The anchor-box-based method requires a large number of anchor boxes to be pre-set, which requires a large amount of computation and is not suitable for learning state supervision tasks. The YOLO series network [13] abandoned the anchor box setting for the first time, which greatly improved the detection speed. The author of LFFD network [14] believes that the receptive field of convolution is a natural anchor box. This method combines SSD’s feature extraction network and YOLO’s detection head to detect faces of different sizes on feature maps of different sizes. Zhang et al. [15] proposed an accurate face detection network based on RetinaNet [12]. The structure of this network is as follows: a backbone composed of ResNet-152 [16] and a six-level Feature Pyramid Network (FPN) neck. Zhu et al. [17] proposed a facial detection network TinaFace, with ResNet-50 [16] and a six-level FPN feature extraction network. CornerNet by Law et al. [18] proposed, for the first time, the use of keypoint prediction to overcome the limitations of anchor boxes, but this method is computationally intensive and has multiple parameters, making it unsuitable for low-performance mobile devices. As an improvement of CornerNet, CenterNet [19] decomposed bounding box prediction into center point prediction and scale prediction, greatly accelerating the network’s running speed. Most of the above networks use feature extraction networks such as Resnet as their backbone, which have large amounts of parameters and computation, and are not suitable for deployment on mobile devices with a low computing performance and small memory. CenterFace [20] used a lighter-weight MobileNet-V2 [21] to extract features based on CenterNet, achieving a better balance between accuracy and speed and achieving real-time face detection on the CPU. However, to deploy on mobile devices with a small memory and weak computing power, the network size still needs to be further reduced. We conducted research on face detection networks based on CenterFace.

### 2.2. Head Pose Estimation

The task of head pose estimation is to detect the three-dimensional Euler angles representing the head orientation: Pitch, Yaw, and Roll. Due to the fact that the algorithm is executed in series with face detection and needs to run on mobile devices, there is a high requirement for running speed. At present, the mainstream head pose estimation methods are mainly divided into facial keypoint-based methods and keypoint-free single-stage methods. Keypoint-based methods [22,23] require first detecting the keypoints of the face and then calculating the pose angle of the head based on the keypoints. This type of method requires a large amount of computation, is slow, and keypoint detection is easily affected by occlusion.

Single-stage methods without keypoints only require RGB or RGB-D images to analyze and obtain the head posture angle. Zhang et al. [24] believe that there is a certain coupling between the three angles representing head posture, and the authors used Center Loss to calculate the pairwise differences between the three angle features. In order to improve the running speed of the network, WHENet [25] used a more lightweight feature extraction network, EfficientNet [26], on the basis of HopeNet [27]. However, the volume of the WHENet network is still large and cannot run in real-time on mobile platforms. Yang et al. [28] proposed FSA-Net, which extracts basic features through a dual-branch feature extraction network using different activation functions and pooling methods. FSA-Net has only 1M parameters and uses lower-resolution input images, greatly improving the network’s running speed. FSA-Net has three shortcomings in feature extraction. Firstly, due to the small size and shallow layers of the network, there is insufficient extraction of deep semantic information. Secondly, the input images of the network are exactly the same, and differences between the two tributaries are only created through activation functions and pooling methods, resulting in weak feature differences between the tributaries. Finally, FSA Net’s fusion of two tributary features through multiplication can easily result in useless features, i.e., zero-value features, covering useful information and causing feature loss. To address these issues, we designed a dual-branch attention feature extraction network.

## 3. Approach

The proposed learning state evaluation method consists of a face detection network, GA-Face, a head pose estimation network, DB-Net, and a learning state evaluation algorithm. GA-Face is responsible for providing the center point coordinates, center point offset, and face width and height of the detected face to DB-Net. DB-Net estimates the posture of the student’s head. The learning state detection algorithm evaluates the learning state of the students based on the distance between their faces and the screen, as well as their head posture.

### 3.1. Face Detection Network: GA-Face

#### 3.1.1. Architecture of GA-Face

**Figure 1:**
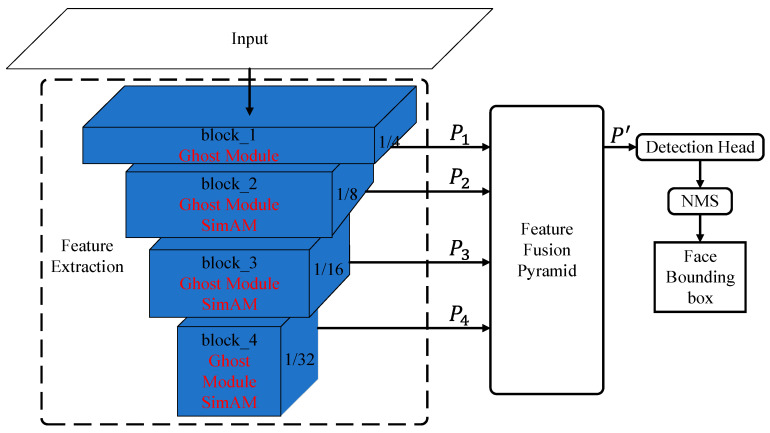
The architecture of GA-Face.

As shown in Figure 1, the anchor-free frame face detection network GA-Face is mainly divided into three parts: the feature extraction network, feature fusion pyramid module, and detection head module. The structure of the feature fusion pyramid module and detection head module is the same as that of CenterFace. In order to reduce the number of parameters in the feature extraction network while ensuring its feature extraction capability, the ghost module [7] and the parameter-free attention module, SimAM [8], were added to the feature extraction module.

The feature extraction network consists of four blocks: block_1, block_2, block_3, and block_4. The four blocks are connected in series and output feature maps P1, P2, P3, and P4 with resolutions of 1/4, 1/8, 1/16, and 1/32 of the original image, respectively. The feature fusion pyramid module fuses P1~P4 and obtains the feature map $P^{\prime }$. The detection head module detects the center point coordinates, center point offset, and face width and height on $P^{\prime }$. After removing duplicate face boxes belonging to the same target through the non-maximum suppression module (NMS), the final result can be obtained.

#### 3.1.2. Feature Extraction Module for Facial Detection Network

Han et al. [7] divided the features obtained from traditional convolution into basic features and redundant features, and they believe that both basic and redundant features are equally important for the network’s expressive power. Due to the similarity between redundant features and basic features, redundant features can be obtained through simple transformations of basic features. Therefore, basic features can be extracted first, and then redundant features can be obtained through group convolution with fewer parameters and less computational complexity. This reduces the computational and parameter complexity of the ghost module proposed by Han et al. by nearly half compared to traditional convolution modules.

**Figure 2:**
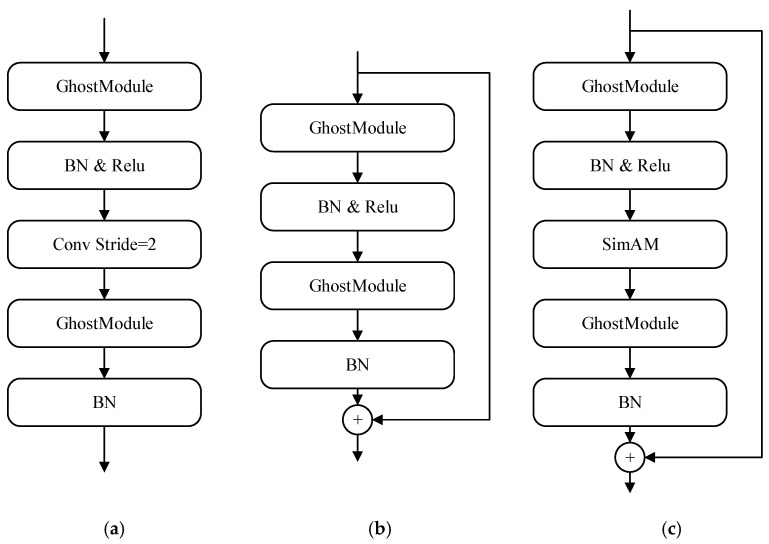
The structure of different network units in feature extraction networks. (**a**) Down-sampling; (**b**) feature extraction; and (**c**) attention enhancement.

Each feature extraction block consists of three basic structures, as shown in Figure 2, namely, the down-sampling structure (Figure 2a), the feature extraction structure with residual links (Figure 2b), and the attention enhancement structure (Figure 2c). Structure (a) is responsible for down-sampling the resolution of the feature map and increasing the number of channels. In the down-sampling structure, the ghost module is first used to increase the number of channels in the feature map to 3–6 times, in order to prevent excessive information loss during the down-sampling process. Then, the down-sampling operation is completed through convolution. Finally, a ghost module is used to reduce the number of channels for feature extraction to 1.5–2 times the initial input. The purpose of conducting this is to recombine the enlarged features, enhance the extraction of semantic information, and reduce the computational and parameter requirements for subsequent feature extraction. Structure (b) is responsible for continuing to extract features. Similar to the down-sampling structure, this structure also uses two ghost modules to increase and decrease the number of channels, respectively. This structure has a residual connection to avoid gradient vanishing during training. Structure (c) is responsible for enhancing the network’s ability to extract important information. This structure embeds a parameter-free attention mechanism, SimAM, between two ghost modules. SimAM will strengthen the important features and suppress useless features based on the features extracted from the first two structures, so that the ghost module below can enhance the impact of the important features on the output when recombining features.

The detailed structure of the feature extraction network is shown in Table 1. In order to ensure that the network can obtain sufficient basic features at the initial stage, traditional convolution is first used in block_1 to extract the basic features from the image. Afterwards, structure (a) is used for another down-sampling operation, and then features are extracted again using structure (b) in the last layer of block_1. Block_1 does not use structures with attention mechanisms, because there is more spatial information in the initial stage of the network and there are fewer feature channels and semantic information, making it difficult for attention mechanisms to function or even have negative effects. In blocks_2 to 4, structure (a) is first used for a down-sampling operation, and then structure (c) is used to enhance the network’s extraction of important features. Due to the fact that block_3 and block_4 already belong to the deeper stages, which contain a large amount of difficult to extract high-level semantic information, a continuous three-layer and two-layer structure (b) are set in block_3 and block_4, respectively, to extract high-level features, and the weight of important features is strengthened through structure (c) at the end.

#### 3.1.3. The Loss Function of Face Detection Networks

The loss of a face detection network is composed of center point classification loss, center point offset loss, and face scale loss [19]. These three losses are responsible for predicting the center point coordinates, center point offset, and face width and height, respectively.

Cross Entropy was used as the center point classification loss.

(1) $$Cross\;Entropy\left(p,y\right)=-\left\{\begin{array}{r} \log(p),\;y=1\\ \log(1-p),\;y=0 \end{array}\right.$$

Among them, $p\in \left[0,1\right]$ represents the predicted value, $y\in \left\{0,1\right\}$ represents the true value label, and y = 1 represents that the sample is the center point of the face. Due to the fact that the number of faces in the image is much smaller than the number of pixels, we use Focal Loss [12] to make the network more focused on training positive samples. The center point classification loss after adding Focal Loss is shown in Formula (2):

(2) $$Focal\;Loss\left(p,y\right)=-\left\{\begin{array}{r} {\left(1-p\right)}^{\gamma }\log(p),\;y=1\\ p^{\gamma }\log(1-p),\;y=0 \end{array}\right.$$

The author of Focal Loss set γ=2. Due to the nature of convolution, each point also contains features of surrounding points, and the predicted value of pixels closer to the center point should be closer to 1. We add center point heat map loss based on Gaussian function to the loss function.

(3) $$M_{ij}=\underset{k=1,2,\ldots K}{\max}e^{-\left(\frac{{\left(i-x_{k}\right)}^{2}}{2\sigma_{w_{k}}^{2}}+\frac{{\left(j-y_{k}\right)}^{2}}{2\sigma_{h_{k}}^{2}}\right)}$$

where (i, j) represent the horizontal and vertical coordinates of each pixel point in the image, $M_{ij}\in \left[0,1\right]$ is the value of each point in the Gaussian heatmap, k represents the number of faces in the image, $\left(x_{k},y_{k}\right)$ represents the coordinates of the center point of the k-th person’s face in the image, $w_{k}$ and $h_{k}$ represent the width and height of the k-th person’s face, respectively, and $\sigma_{w_{k}}^{2}$ and $\sigma_{h_{k}}^{2}$ are adaptive variances related to the scale of the k-th person’s face. Due to the possibility of overlapping Gaussian heatmaps of different faces, it is necessary to determine the values of each point on the entire Gaussian heatmap using max.

The Gaussian heatmap M increases the probability of predicting pixels closer to the center as positive samples, which suppresses the loss of negative samples closer to the center. The center point classification loss with the addition of a Gaussian heat map is shown in Formula (4) as follows:

(4) $$L_{center}\left(p,y,M\right)=-\frac{1}{K}\sum_{i=1}^{W/4}\sum_{j=1}^{H/4}\left\{\begin{array}{r} {\left(1-p_{ij}\right)}^{\gamma }\log(p_{ij}),\;y=1\\ {\left(1-M_{ij}\right)}^{\alpha }{p_{ij}}^{\gamma }\log(1-p_{ij}),\;y=0 \end{array}\right.$$

We set hyperparameters α=4. Due to the error caused by zooming in the bounding box back to the original image, which is four pixels in both the horizontal and vertical directions, it is necessary to add center point offset prediction to compensate for the lack of fine-grained information. We map the offset within four pixels to the interval [0,1]. The center point offset loss is shown in Formula (5):

(5) $$L_{offset}\left(\hat{o},o\right)=\frac{1}{K}\sum_{k=1}^{K}SmoothL1\left({{\hat{o}}_{k},o}_{k}\right)$$

Among them, $\hat{o}$ is the predicted value of the center point offset, o is the true value label, and negative samples are 0.

The facial scale loss function is shown in Formula (6):

(6) $$L_{scale}\left(\hat{s},s\right)=\frac{1}{K}\sum_{k=1}^{K}SmoothL1\left({{\hat{s}}_{k},s}_{k}\right)\;s=\log\left(\frac{s_{0}}{4}\right)$$

where $\hat{s}$ is the predicted value of the center point offset and $s_{0}$ is the true value label. As the network completes the prediction on a feature map down-sampled by four times, the scale value needs to be divided by four.

The total loss function of the network is obtained by weighting three losses, and the total loss is shown in Formula (7) as follows:

(7) $$L=\lambda_{c}L_{center}+\lambda_{o}L_{offset}+\lambda_{s}L_{scale}$$

where $\lambda_{c}$, $\lambda_{o}$, and $\lambda_{s}$ are the weights of the three losses, respectively. We determined the weights of the three losses based on their importance, but the specific values for each weight are set based on experience. Since center point prediction is the main task of the network, its weight $\lambda_{c}$ is set to 1.0. The center point offset loss has a relatively small impact on the results, so its weight $\lambda_{o}$ is set to 0.1. Due to the small range of facial size changes for students during online learning, the face scale loss $\lambda_{s}$ is set to 0.01.

### 3.2. Head Pose Estimation Network: DB-Net

**Figure 3:**
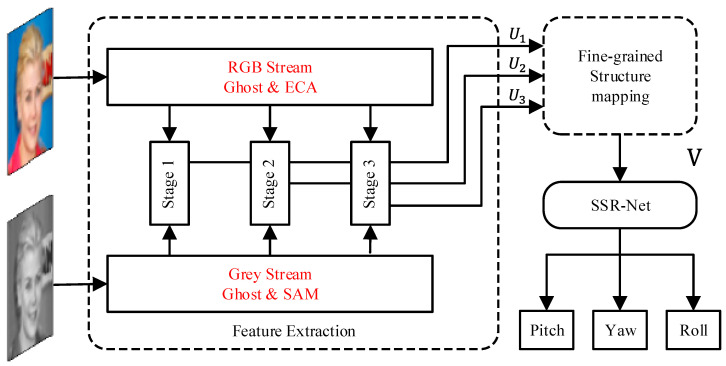
The architecture of DB-Net.

The architecture of the proposed DB-Net is shown in Figure 3. The network consists of a dual-branch feature extraction network, a fine-grained structure mapping module, and a soft-stage regression (SSR) module. The structure of the fine-grained structure mapping module and SSR module is the same as that of FSA-Net [28].

**Figure 4:**
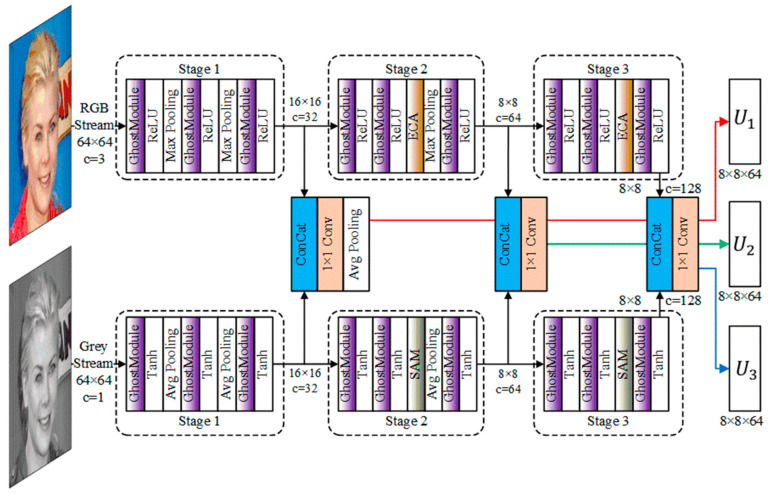
The structure of a dual-branch feature extraction network.

As shown in Figure 4, the dual-branch feature extraction network consists of two feature extraction branches, an RGB branch and grayscale branch. Each branch is divided into three stages, and three ghost modules are used to extract features in each stage. The ReLU activation function and maximum pooling method are used in the RGB branch, and the Tanh activation function and average pooling method are used in the grayscale branch. Efficient Channel Attention (ECA) [29] was added in Stages 2 and 3 of the RGB tributaries. Spatial Attention Mechanisms (SAM) [30] were added in Stages 2 and 3 of the gray tributaries. Use 1 × 1 convolution to connect the features of the corresponding stages of the two tributaries. The convolutional fusion operation ultimately obtains three sets of multi-scale features of consistent size: U1, U2, and U3. RGB color tributaries have three types of color channel information, and the combination of different channels can express different types of features, such as texture, color, and contour. Grayscale tributaries do not have complex color effects and can better display spatial regional relationships. We replaced the traditional convolution module in the feature extraction branch with the ghost module. The ghost module divides traditional convolution into two concatenated small convolution modules. Although the two convolution modules have different functions and structures, they can still increase the depth and receptive field range of the network. DB-Net connects the features of two tributaries through 1 × 1 convolution, allowing the network to autonomously learn how to fuse different types of features.

### 3.3. Learning State Evaluation Algorithm

Face detection can obtain facial frames. The size of the face frame can reflect the distance between the student’s head and the screen. When students listen attentively, the area of the face frame will also increase. When students start to lose focus, they may lean away from the screen and rest, resulting in a smaller face frame area. Therefore, the area of the facial frame within the camera’s field of view can reflect the student’s learning status. If the area of the facial frame is less than the threshold A, it is considered that the student’s head is too far away to see the screen clearly, indicating that they are in a distracted state. When learning through mobile devices, the optimal distance for line of sight is about 40–60 cm. However, when the distance exceeds 100 cm, it is already difficult for students to see the screen content clearly. At this time, the student’s facial frame area is about 1/4 of the optimal distance. Therefore, we set the threshold A to 0.25, which means that, when the facial frame area becomes 25% of the benchmark, it is considered to be distracted.

The head posture can obtain the three-dimensional Euler rotation angle of the student’s head, which is the orientation of the student’s head. When people focus on things, they always have their heads facing the focal point of attention. Therefore, by analyzing the head posture, it is possible to determine whether the student is staring at the screen. Students may experience a certain range of changes in their head orientation when looking at different positions on the screen. For example, when reading content at the edge of the screen, students may not be paying attention to the central area, and their heads may be facing left or right, not completely facing the screen, but they are indeed listening attentively. Therefore, students may be in a serious listening state when their heads are facing within a certain angle range. We use Gaussian probability to determine the probability that students are paying attention to the content on the screen.

(8) $$P=G_{ij}=e^{-\left(\frac{{\left(i-x\right)}^{2}}{2\sigma_{w}^{2}}+\frac{{\left(j-y\right)}^{2}}{2\sigma_{h}^{2}}\right)}$$

where (i, j) represent the coordinates of the screen center point in the camera coordinate system, (x, y) represent the coordinates of the student’s head facing a projection point on the camera plane, which is the focus of attention, w and h are the width and height of the effective field of view projected on the camera plane with the student’s head facing as the center, and $\sigma_{w}$ and $\sigma_{h}$ are the coefficients related to w and h, used to control the distribution of Gaussian probability. We set $\sigma_{w}=0.2w$ and $\sigma_{h}=0.2h$.

The range of probability P is (0,1), and the student’s attention score S is calculated using the following formula.

(9) S=100×P

During the learning process, students may experience constant changes in their head orientation, but they are indeed listening attentively. For example, when taking notes, students may switch their heads back and forth between the screen and the notebook, which can easily be mistaken as distraction. In order to reduce false positives, the algorithm records data from the previous 10 s during detection and calculates the weighted average of attention scores $\bar{S}$ within 10 s using Formula (10)

(10) $$\bar{S}=\frac{\sum_{k=1}^{K}S\left(k\right)\times \frac{k}{K}}{\sum_{k=1}^{K}\frac{k}{K}}$$

where K is the number of detections recorded within 10 s, S (k) is the attention score of the kth record in chronological order, and k/K is the weight assigned to each time step. The closer the data are to the current time step, the greater the weight. Finally, a threshold of 60 points is set as the attention score. When the attention score $\bar{S}$ is below 60 points, it is considered that the student’s attention level is too low and they need to be reminded by the teacher.

**Figure 5:**
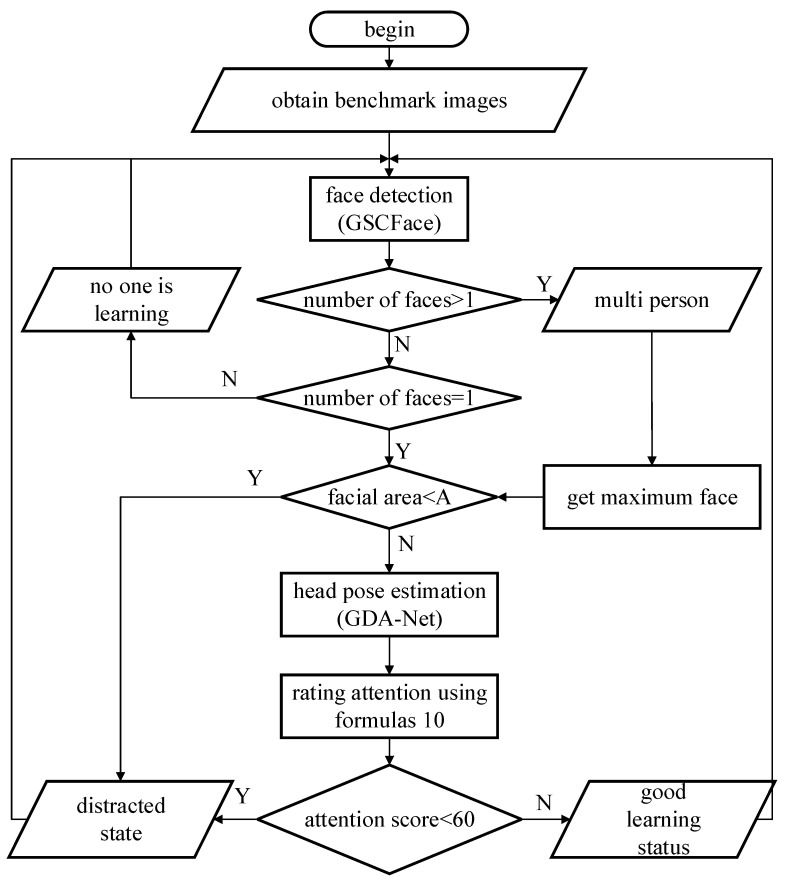
The process of student learning state algorithm.

The process of the student learning state evaluation algorithm is shown in Figure 5. The evaluation algorithm can categorize the learning state of students into four states: nobody, learning in progress, multiplayer, normal, and distracted. When the face detection network does not detect a face, it is judged as nobody. When the face detection network detects two or more faces, it indicates that there are other people around the student, which may affect their learning efficiency and be determined as a multi-person state. The person with the largest facial frame area is a student who is currently studying. We can calculate the student’s attention score based on Formula (10). If a student’s attention score is above 60, it is considered to be normal learning, and if it is below 60, it is considered to be distracted. When the area of the detected student’s facial frame is less than the threshold, it indicates that the student is too far away from the screen, judged as a distracted state, and the current attention score is directly set to 0.

## 4. Experiment

### 4.1. Effectiveness Evaluation of Face Detection Network GA-Face

We tested the performance of the proposed GA-Face on the mainstream face detection datasets FDDB [31] and WiderFace [32]. FDDB (Face Detection Data Set and Benchmark) contains 2845 images, each containing at least one person’s face, resulting in a total of 5171 faces.

The WiderFace dataset contains 12,280 images and 158,989 faces, which vary greatly in size, expression, posture, occlusion, lighting, and other aspects. WiderFace divides the validation set into three detection difficulties: Easy, Medium, and Hard. Easy has large faces with less occlusion, Medium has relatively small and partially occluded faces, and Hard has small and blurry faces. The detection ability of the network can be accurately evaluated through different difficulty levels of classification.

We used the training set of WiderFace for training and tested it on the FDDB dataset and WiderFace validation set. Considering the actual usage environment of the network, the camera resolution and focus of devices cannot be consistent, and many low-performing devices have cameras that are prone to noise and image quality damage. Therefore, this article expands the training data during training. The main data expansion methods include: random scaling, random translation, random cropping, random noise, random sharpening, random flipping, color jitter, random brightness, and random contrast. After expansion, the images are uniformly scaled to a resolution of 800 × 800.

The performances of face detection networks are evaluated using two metrics: Average Precision (AP) and Frames Per Second (FPS). We list the mean Average Precision (mAP) for the WiderFace dataset. GA-Face is trained for 120 epochs on the WiderFace training set, the network weights are optimized using the Adam optimizer, a learning rate of 0.0005 is achieved, and the learning rate is reduced by 10 times when training reaches the 70th and 100th epochs. The operating system used in this article is Windows 10, with Intel Xeon E5-2696v2 CPU, NVIDIA GeForce 2080 GPU, Python version 3.8, Python version 1.8.1, and CUDA version 11.1. The phone model used for testing FPS is Huawei Mate 20 Pro, and the CPU of the phone is Huawei Kirin 980.

We conducted comparative experiments on RetinaFace [33], LFFD [13], CenterFace [20], ASFD [34], and the proposed GA-Face network on the FDDB dataset and WiderFace validation set. In Table 2, GA-Face (ghost only) represents removing the SimAM module from the attention enhancement structure (Figure 2c) of GA-Face. This can verify the effectiveness of the ghost module added to GA Face. Similarly, GA-Face (SimAM only) in Table 2 represents removing the ghost module from the three structures shown in Figure 2. This can verify the effectiveness of the SimAM module added to GA-Face. The experimental results are shown in Table 2:

(1) RetinaFace has a higher accuracy than CenterFace on both datasets, but due to its approximately 100,000 pre-set anchor boxes, the network running speed is only 16FPS, making it unable to achieve real-time face detection on mobile devices.
(2) The average accuracy of the GA-Face network on the FDDB dataset is 1.3% higher than CenterFace and 0.6% higher than RetinaFace. The accuracy of GA-Face on WiderFace’s Easy and Medium difficulty is on par with CenterFace, while it is slightly lower on Hard difficulty. This is because the ghost module sacrifices some basic features, reducing its ability to detect extremely small and blurry faces, but this does not affect the network’s use in online learning scenarios. The network parameter count of GA-Face is 55% less than that of CenterFace. The FPS of GA-Face doubles compared to RetinaFace and increases by 32% compared to CenterFace. The network can reach a speed of 24FPS when deployed on mobile devices, achieving real-time detection results.
(3) ASFD has multiple versions, with ASFD-D0 designed for mobile devices and ASFD-D6 being the most comprehensive version. The ASFD-D6 version has the highest accuracy, but it has the highest number of parameters and the lowest inference speed. As a mobile-device-specific version, ASFD-D0 has the fastest speed, but its accuracy is lower than GA-Face.
(4) The accuracy of GA-Face using only the ghost module is lower than that of GA-Face on both datasets. The accuracy of using only SimAM’s GA-Face is similar to that of GA-Face, but the speed decreases. SimAM has a slight impact on the speed of the network, but has a positive impact on the accuracy of the network. GA-Face achieves a good balance between speed and accuracy.

**Figure 6:**
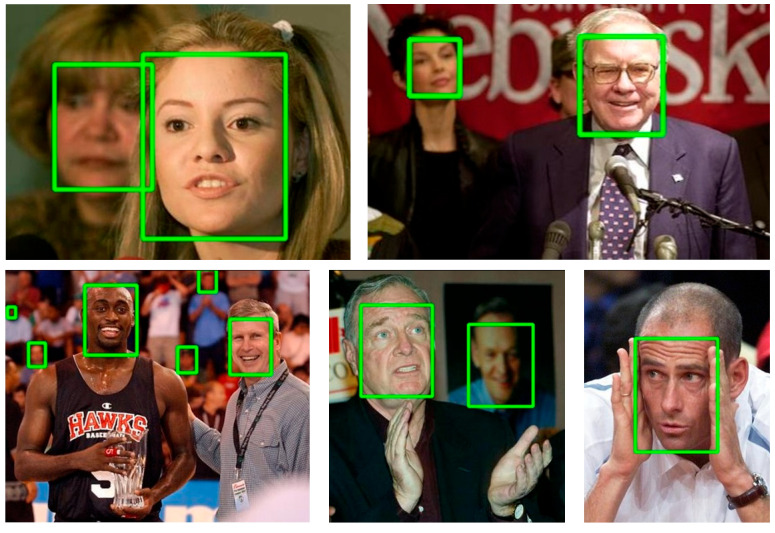
The face detection performance of GA-Face on FDDB dataset.

**Figure 7:**
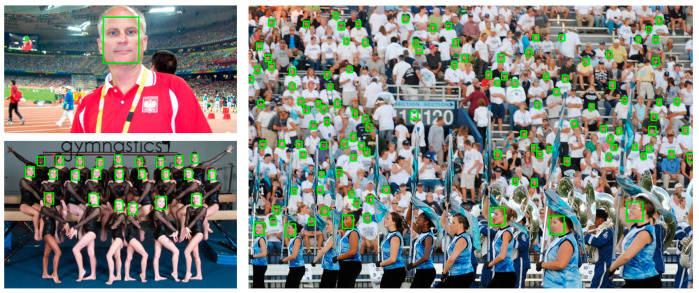
The face detection performance of GA-Face on WiderFace validation set.

Figure 6 and Figure 7 show the detection results of the GA-Face network on the FDDB dataset and WiderFace dataset. The network performs very well in detecting large and medium-sized faces, but its ability to detect small and blurry faces is average. In online learning scenarios, the faces of students are usually larger, and the GA-Face network is suitable for the application scenario of this article.

### 4.2. Effectiveness Evaluation of Head Pose Estimation Network DB-Net

In this section of the experiment, GA-Face was first used to detect a single person’s face. Then, the face frame was enlarged by 1.3 times and adjusted to a square according to the longest side to ensure that the cropped image could accommodate the entire head of the target. After cropping the face, the data were expanded using methods such as random scaling, random sharpening, random brightness, random contrast, and color jitter. Finally, the image resolution was uniformly scaled to 64 × 64.

We evaluated DB-Net on three datasets: 300W-LP, AFLW2000 [35], and BIWI [36]. We trained the network on the 300W-LP dataset and tested its accuracy on the AFLW2000 and BIWI datasets, as well as its running speed on the CPU. The trained DB-Net was deployed on a mobile phone to test FPS. The experiment used the Adam optimizer to update the network weights, with an epoch of 90 and an initial learning rate of 0.001. When training reached 30 and 60 epochs, the learning rate decreased by 10 times. The operating system used in this experiment was Windows 10, with Intel Xeon E5-2696v2 CPU, NVIDIA GeForce 2080 GPU, Python version 3.8, Python version 1.8.1, and CUDA version 11.1.

We compared EVA-GCN [23], HopeNet [27], WHENet [25], FSA-Net [28], and the DB-Net proposed in this paper. Table 3 shows the Absolute Error (AE) and Mean Absolute Error (MAE) of the three angles of head posture, while Table 4 shows the inference speed FPS and the total number of network parameters. We conducted ablation studies on the ghost module and ECA&SAM module added to DB-Net in Table 3 and Table 4. The head pose estimation network without the ghost module and ECA&SAM module in Figure 4 is represented as DB-Net (ECA&SAM only) and DB Net (ghost only) in Table 3 and Table 4, respectively. In Table 3, the values below the pitch, yaw, and roll columns represent the AE values for each method, while the values under the MAE column represent the MAE values for each method.

(1) The accuracy of DB-Net (ECA&SAM only) is slightly higher than that of FSA-Net, indicating that both ECA and SAM can enhance the network’s feature extraction ability. However, ECA and SAM also slightly increase the number of network parameters, slightly affecting the running speed.
(2) DB-Net (ghost only) has a slightly higher accuracy than FSA-Net, indicating that the ghost module deepens the network and increases the receptive field, which has a positive impact on accuracy. At the same time, the Ghost module reduces the number of parameters and computation, and the network speed is improved.
(3) On two datasets, the AE of DB-Net decreases by 0.36 compared to FSA Net, the number of network parameters decreases by 27%, and the running speed increases by 16%. The network speed on mobile phones can reach 126FPS, meeting the requirements of real-time operation.

**Figure 8:**
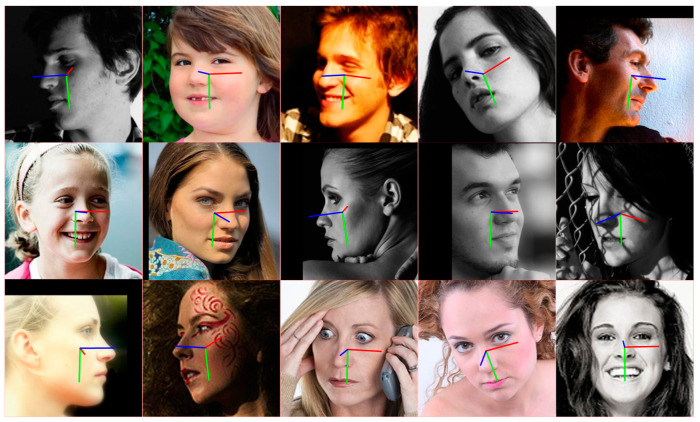
The effectiveness of head pose estimation network DB-Net.

Figure 8 shows the detection results of DB-Net on AFLW2000. From the perspective of detection performance, the network performs well with different environments, expressions, and postures.

### 4.3. Evaluation of the Effectiveness of Learning State Evaluation Algorithms

We developed a learning state assessment app using the Qt for the Android development environment and deployed the trained network model at the mobile end. The experiment tested the execution time and FPS of each step of the entire learning state evaluation algorithm on a mobile phone, and compared the speed impacts of different resolution images on the algorithm. The experimental results are shown in Table 5:

From the results shown in Table 5, it can be seen that the resolution of the input image only affects the speed of the GA-Face. At a resolution of 320 × 240, the speed of the face detection network is faster than a 640 × 480 resolution increased by 10FPS. Therefore, we use 320 × 240 resolution as the input resolution, and the evaluation algorithm is executed every other frame, with a camera frame rate of 30FPS and an algorithm execution rate of 15FPS.

The proposed GA-Face and DB-Net adopt the frameworks of CenterFace and FSA-Net, respectively. The baseline method uses CenterFace for face detection and FSA Net for head pose estimation. We tested the baseline inference speed on a 320 × 240-pixel image captured by our mobile phone. In Table 6, our method uses GA-Face and DB-Net for face detection and head pose estimation, respectively. From Table 6, it can be seen that the proposed method outperforms the baseline method in terms of inference speed and is more suitable for running on mobile devices with insufficient computing power.

**Figure 9:**
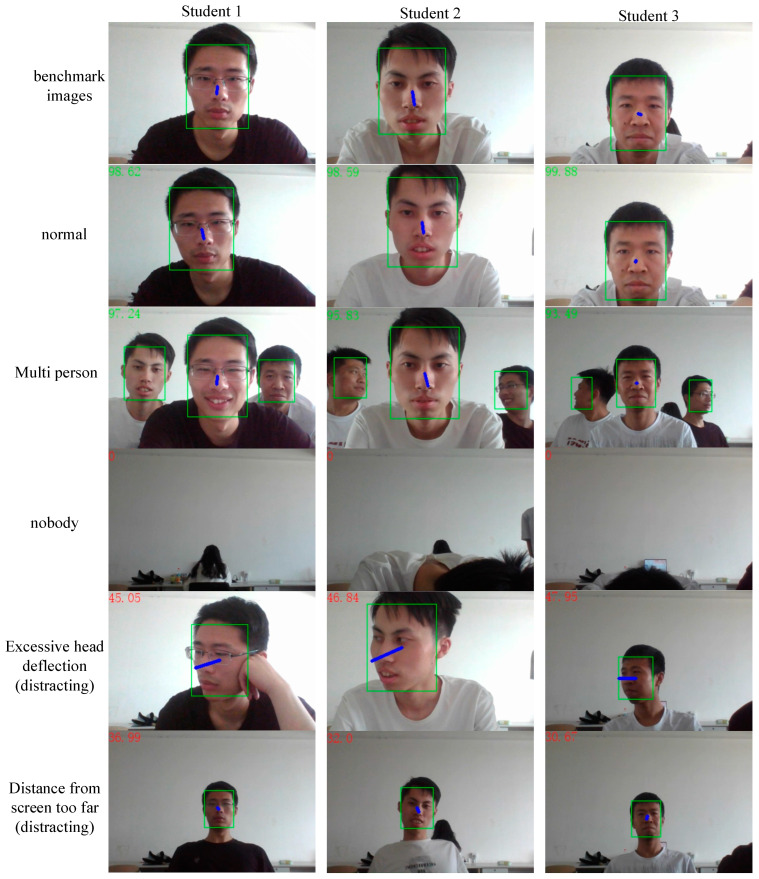
Assessment results of the learning status of three students.

**Figure 10:**
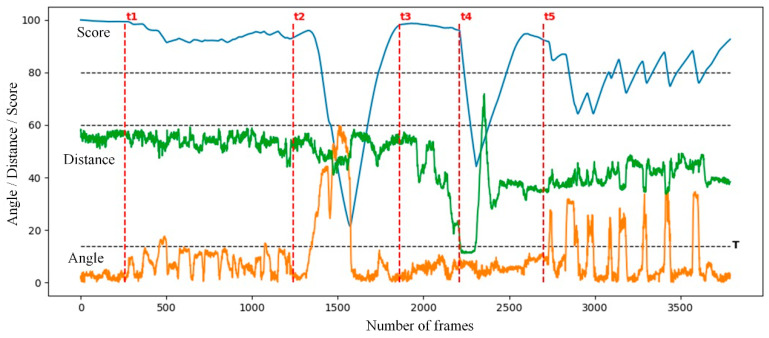
The curve of angle, distance, and fraction versus frame rate.

Figure 9 shows the changes in the facial frame area, head deflection angle, and attention score of three students participating in the test. The number in the upper left corner of each image is the attention score. The blue, green, and yellow curves in Figure 10 show the changes in attention score, facial frame area, and head deflection angle, respectively.

Before time t1, the area of the student’s facial frame did not change by more than 15%, and the head deflection angle did not exceed 5°. The algorithm’s attention rating for the student remained stable at around 99 points. During the time period from t1 to t2, the students were reading the content presented by the teacher. Therefore, the fluctuation range of the facial frame area was about 30%, and the fluctuation range of the head deflection angle was about 20°. Although the attention score slightly decreased, it still exceeded 90 points, indicating a normal learning state. During the time period from t2 to t3, the students were distracted by other things and their head deflection angle gradually increased to 60°, resulting in a rapid decrease in attention scores to below 60 points. At time t4, the student leaned back due to fatigue, and the face frame area decreased by 15% of the baseline, below the threshold. At this point, the student could no longer see the screen content clearly, and the algorithm determined them to be in a distracted state. After time t5, the student began to take notes, and the deviation angle of their head caused a rapid fluctuation of about 35°, causing their attention score to fluctuate back and forth between 70 and 90 points, indicating a normal learning state. This indicates that the algorithm can effectively avoid the occurrence of misjudgments when students are taking notes and other normal learning states that require a small shift in focus.

## 5. Conclusions

The experimental results indicate that the overall computational complexity of the proposed online learning state evaluation method based on face detection and head pose estimation is relatively low. This can ensure that it will not disturb the normal learning process of students. The proposed face detection network, GA-Face, and head pose network, DB-Net, achieve a good balance in accuracy and computational complexity. The proposed online learning state evaluation method can visually display the degree of student attention concentration and reduce the occurrence of misjudgments through attention scores.

In the proposed learning state evaluation method, a fixed threshold A is used. Considering that students of different ages and heights should have different comfortable reading distances, in the next step of this research, we will design adaptive thresholds based on user reading habits.

## Figures and Tables

**Table 1:** Detailed structure of feature extraction network.

	Input Resolution	Convolutional Kernel Size	Input Channels	Intermediate Channel	Output Channel	Composition Structure
block_1	224 × 224	3	3	/	16	Conv
	112 × 112	3	16	48	24	(a)
	56 × 56	3	24	72	24	(b)
block_2	56 × 56	3	24	72	40	(a)
	28 × 28	3	40	120	40	(c)
block_3	28 × 28	3	40	240	80	(a)
	14 × 14	3	80	200	80	(c)
	14 × 14	3	80	184	80	(b)
	14 × 14	3	80	184	80	(b)
	14 × 14	3	80	480	112	(b)
	14 × 14	3	112	672	112	(c)
block_4	14 × 14	3	112	672	160	(a)
	7 × 7	3	160	960	160	(c)
	7 × 7	3	160	960	160	(b)
	7 × 7	3	160	960	160	(b)
	7 × 7	3	160	960	160	(c)

**Table 2:** Performance comparison of face detection networks.

Method	FDDB (AP%)	WiderFace Easy (AP%)	WiderFace Medium (AP%)	WiderFace Hard (AP%)	WiderFace (mAP%)	FPS (CPU)	FPS (Phone)	Parameters (×10^6^)
RetinaFace	95.2	90.7	90.1	87.4	89.4	16	-	0.73
LFFD	93.2	85.8	82.1	74.1	80.7	21	-	2.15
ASFD-D6	98.1	95.8	94.7	86.0	92.2	5	-	8.61
ASFD-D0	92.4	90.1	87.5	74.4	84.0	36	28	0.68
CenterFace	94.5	88.1	87.5	82.5	86.0	25	18	1.80
GA-Face (Ghost only)	92.7	85.2	84.4	78.7	82.8	35	25	0.81
GA-Face (SimAM only)	94.9	88.6	87.9	81.9	86.1	24	18	1.80
GA-Face	95.8	88.3	87.3	81.0	85.5	33	24	0.81

**Table 3:** MAE of each network on the AFLW2000 and BIWI datasets.

Method	AFLW2000				BIWI			
	Pitch	Yaw	Roll	MAE	Pitch	Yaw	Roll	MAE
EVA-GCN	5.97	4.38	4.23	4.86	5.92	4.53	3.37	4.60
HopeNet	6.56	6.47	5.44	6.16	6.60	4.81	3.27	4.90
WHENet	5.76	4.35	4.29	4.80	5.44	4.38	3.30	4.37
FSA-Net	6.22	4.34	4.65	5.07	6.46	4.83	3.56	4.95
DB-Net(ECA&SAM only)	5.79	4.16	4.52	4.82	6.18	4.95	3.60	4.91
DB-Net(Ghost only)	5.89	4.22	4.47	4.86	5.97	5.02	3.45	4.81
DB-Net	5.83	4.09	4.22	4.71	5.64	4.83	3.31	4.59

**Table 4:** FPS and parameter quantity of each network.

Method	FPS (CPU)	FPS (Cell Phone)	Parameters (×10^6^)
EVA-GCN	56	-	6.22
HopeNet	22	-	23.9
WHENet	119	52	4.40
FSA-Net	288	98	0.29
DB-Net DB-Net (ECA&SAM only)	265	96	0.31
DB-Net(Ghost only)	356	134	0.19
DB-Net	336	126	0.21

**Table 5:** Execution time and FPS of each module of the algorithm.

Method	Inference Time (640 × 480)	FPS (640 × 480)	Inference Time (320 × 240)	FPS (320 × 240)
Face detection (GA-Face)	41 ms	24	29 ms	34
Head pose estimation (DB-Net)	8 ms	126	8 ms	126
Learning state evaluation	3 ms	384	3 ms	384
Total	52 ms	19	40 ms	25

**Table 6:** Comparison of inference speed.

Method	Inference Time	FPS
Ours	37 ms	25
Baseline	54 ms	18

## Data Availability

Data are contained within the article and references.
